# MITIG.RA: study protocol of a tailored psychological intervention for managing fatigue in rheumatoid arthritis randomized controlled trial

**DOI:** 10.1186/s13063-023-07692-4

**Published:** 2023-10-06

**Authors:** Cátia Duarte, Ruben L. F. Spilker, Cláudia Paiva, Ricardo J. O. Ferreira, José A. Pereira da Silva, Ana M. Pinto

**Affiliations:** 1 Rheumatology Department, Centro Hospitalar E Universitário de Coimbra, Coimbra, Portugal; 2 Faculty of Medicine, Coimbra Institute for Clinical and Biomedical Research (iCBR), University of Coimbra, Coimbra, Portugal; 3 Faculty of Medicine, University of Coimbra, Coimbra, Portugal; 4 Nursing Research, Innovation and Development Centre of Lisbon (CIDNUR), Nursing School of Lisbon (ESEL), Lisbon, Portugal; 5 Centre for Research in Neuropsychology and Cognitive and Behavioural Intervention (CINEICC), Faculty of Psychology and Educational Sciences, University of Coimbra, Coimbra, Portugal; 6 Institute of Psychological Medicine, Faculty of Medicine, University of Coimbra, Coimbra, Portugal

**Keywords:** Rheumatoid arthritis, Fatigue, Psychotherapy, Mindfulness, Self-compassion, Acceptance and commitment therapy

## Abstract

**Background:**

Despite remarkable medical advances in the treatment of rheumatoid arthritis (RA), a subset of patients fails to achieve complete clinical remission, as the Patient Global Assessment (PGA) of disease activity remains above 1, even after the inflammatory process is brought under control. This so-called state of ‘PGA-near-remission’ negatively impacts individuals’ functioning and potentiates inadequate care. Fatigue is a distressing and disabling symptom frequently reported by patients in PGA-near-remission, and its management remains challenging. While classic cognitive-behavioural interventions show some benefits in managing fatigue, there is potential for improvement. Recently, contextual-cognitive behavioural therapies (CCBT), like mindfulness, acceptance, and compassion-based interventions, have shown promising results in fatigue-associated disorders and their determinants. This study primarily aims to examine the efficacy of the Compassion and Mindfulness Intervention for RA (MITIG.RA), a novel intervention combining different components of CCBT, compared to treatment-as-usual (TAU) in the management of RA-associated fatigue. Secondary aims involve exploring whether MITIG.RA produces changes in the perceived impact of disease, satisfaction with disease status, levels of depression, and emotion-regulation skills.

**Methods:**

This is a single center, two-arm parallel randomized controlled trial. Patients will be screened for eligibility and willingness to participate and will be assessed and randomized to the experimental (MITIG.RA + TAU) or control condition (TAU) using computer randomization. MITIG.RA will be delivered by a certified psychologist and comprises eight sessions of 2 h, followed by two booster sessions. Outcomes will be assessed through validated self-report measures, including fatigue (primary outcome), perceived impact of disease, depressive symptoms, mindfulness, self-compassion, safety, and satisfaction (secondary outcomes). Assessment will take place at baseline, post-intervention, before the first and second booster sessions (weeks 12 and 20, respectively), and at 32 and 44 weeks after the interventions’ beginning.

**Discussion:**

We expect MITIG.RA to be effective in reducing levels of RA-associated fatigue. Secondarily, we hypothesize that the experimental group will show improvements in the overall perceived impact of disease, emotional distress, and emotion regulation skills. Our findings will contribute to determine the benefits of combining CCBT approaches for managing fatigue and associated distress in RA.

**Trial registration:**

ClinicalTrials.gov NCT05389189. Registered on May 25, 2022.

## Administrative information

Note: The numbers in curly brackets in this protocol refer to the SPIRIT checklist item numbers. The order of the items has been modified to group similar items

**Table:** 

Title {1}	MITIG.RA: study protocol of a tailored psychological intervention for managing fatigue in rheumatoid arthritis randomized controlled trial
Trial registration {2a and 2b}	ClinicalTrials.gov NCT05389189. Registered on May 25, 2022, before the start of inclusion.
Protocol version {3}	Protocol version 2, May 1, 2022
Funding {4}	*The study has no monetary funding*
Author details {5a}	Cátia Duarte*, ^1,2^ Ruben L.F. Spilker,*^3^ Cláudia Paiva,^1^ Ricardo J.O. Ferreira,^4^ José A. Pereira da Silva,** ^1,2^ Ana M. Pinto**,^5,6^ Affiliations ^1^ Rheumatology Department, Centro Hospitalar e Universitário de Coimbra, Portugal. ^2^ Coimbra Institute for Clinical and Biomedical Research (iCBR), Faculty of Medicine, University of Coimbra, Portugal ^3^Faculty of Medicine, University of Coimbra, Portugal. ^4^ Nursing Research, Innovation and Development Centre of Lisbon (CIDNUR), Nursing School of Lisbon (ESEL), Portugal ^5^ Centre for Research in Neuropsychology and Cognitive and Behavioural Intervention (CINEICC), Faculty of Psychology and Educational Sciences, University of Coimbra, Portugal. ^6^ Institute of Psychological Medicine, Faculty of Medicine University of Coimbra, Portugal. * and **Contributed equally to this paper
Name and contact information for the trial sponsor {5b}	*The study has no sponsor*
Role of sponsor {5c}	*The study has no sponsor*

## Introduction

### Background and rationale {6a}

Rheumatoid arthritis (RA) is a chronic condition of unknown aetiology, characterized by pain, swelling, stiffness, and progressive disability, caused by the inflammation and gradual destruction of the synovial joints [1]. It affects around 0.5% of the adult population worldwide, being more frequent in women [2, 3], and significantly impacts several health-related domains (e.g. fatigue, sleep), affecting the individual’s functioning, well-being, and quality of life [4–6].

Current recommendations on treatment and management of RA follow a treat-to-target (T2T) approach aimed at achieving remission of the inflammatory process, or at least low disease activity, as soon and as consistently as possible [7, 8]. The guiding principles include regular quantitative assessments of inflammatory activity and consequent adjustment of immunosuppressive medication to ensure persistent clinical and analytical remission [8]. The definitions of remission currently endorsed by the American College of Rheumatology (ACR) and the European Alliance of Associations for Rheumatology (EULAR) [9], are based on tender and swollen joint counts, acute phase reactants, and the patients’ global assessment of disease activity (PGA), with or without the physician’s global assessment [9].

Due to advances in pharmacological treatments and the implementation of the T2T approach, remission has become a feasible and rather frequent outcome in RA [10]. However, about one-third of all patients with RA fail to achieve a complete remission status according to the Boolean definition endorsed by ACR and EULAR, despite the absence of inflammation, solely due to the PGA score (> 1), a status coined ‘PGA near-remission’ [11–13]. This condition entails an elevated risk of overtreatment if current recommendations are strictly followed [14–16]. These patients’ conditions cannot be improved by additional immunosuppression, as the disease process is already under control. Instead, they require adjunctive measures designed to address the mechanisms underlying the unabated PGA, with emphasis on fatigue [12].

Published reports suggest that pain, functional impairment, fatigue, and comorbidities (e.g. depression and anxiety) are the major determinants of PGA [12, 13, 17], especially after remission of the inflammatory process has been achieved [12, 17]. In this sense, PGA is primarily a measure of disease impact, i.e. a mirror of current somatic symptoms and functional impairment, rather than a reliable reflexion of inflammatory activity [12, 17].

These observations support the proposal for a dual target strategy in the management of RA [16] whereby a specific target focused on the patient’s experience of the disease would be pursued in parallel with the current one, defined by disease remission, sharpened by the exclusion of PGA.

In RA, more than 70% of patients report levels of fatigue that are similar to those observed in chronic fatigue syndrome [18], and half of all patients report it as severe and of the highest priority in their list of persistent complaints [19]. These observations are similar among patients in PGA-near-remission despite being virtually devoid of joint inflammation [12]. Patients often consider that this symptom is unduly ignored by clinicians [20].

Multiple factors have been associated with fatigue observed in RA [21], reinforcing its complex and multicausal nature [22]. Depression, disability/inactivity, and sleep disturbances seem to be key drivers of fatigue [23], whereas pain and disease activity appear to play a rather minor role [22]. This complexity underlies the major challenge faced so far in the design and implementation of effective interventions.

Adjunctive non-pharmacological interventions, including exercise, counselling, occupational therapy, cognitive behavioural therapy (CBT), and other psychological interventions, have been associated with positive results [24–26]. CBT is the gold standard intervention in many mental and physical health conditions, and a valuable intervention in RA [27–29], with proven beneficial effects in chronic pain, sleep difficulties, fatigue, self-management, self-care, coping, and well-being, both in individual and group settings [30].

Lately, contextual CBT interventions, such as mindfulness, acceptance, and compassion-based approaches, have equally been showing promising results [31, 32]. These types of interventions promote the establishment of a different relationship with internal experiences (e.g. cognitions and emotions) and the body, variables of vital importance in the experience and management of RA [33]. Preliminary evidence suggests that mindfulness-based interventions may help improve RA-related outcomes and associated psychological distress [31, 34, 35]. Regarding acceptance and commitment therapy (ACT), studies have found it effective in enhancing the quality of life [36]; improving chronic illness-related symptoms, such as pain, depression, and anxiety [37–39]; and decreasing fatigue levels in chronic fatigue syndrome [40]. ACT has also been shown to implicitly promote self-compassion (e.g. a warm and compassionate stance towards oneself in the face of setbacks [41], a recognized buffer against depression and negative pain-related outcomes [42]). Although CBT and ACT differ in their philosophical stance and therapeutic techniques [43, 44], they can be integrated in a coherent way in order to target different processes (i.e. pain, sleep disturbance, emotion dysregulation, psychological and behavioural inflexibility, functional impairment) underlying the maintenance and exacerbation of physical and mental symptoms, including fatigue, in RA [45]. The integration of traditional CBT and mindfulness, acceptance, and compassion-based approaches reflects the expansion of the field, in the face of cumulative evidence, the inclusion of both change-oriented and acceptance-oriented strategies in context-sensitive approaches, and follows efforts to optimize existing CBT protocols to favour or improve treatment response [46]. In addition, a combination of different contextual cognitive-behavioural interventions, such as those included in the MITIG.RA programme (i.e. mindfulness, acceptance, and compassion-based strategies), have been previously proposed and tested in people with binge eating disorder [47], cancer [48], chronic pain [49], and overweight and obesity [50]. Overall, these studies have shown promising results in improving disease-specific symptoms, emotional distress, cognitive and emotional regulation, healthy behaviours, social functioning, and quality of life.

The intervention and therapeutic techniques used in this study are feasible, accepted, and validated in online environments [51–53] and reveal encouraging outcomes in other chronic illnesses [42, 48, 53]. The researchers combine distinct evidence-based therapeutic components in a complementary and coherent way to target relevant processes at play in RA with the objective of alleviating fatigue in patients with this condition.

### Objectives {7}

The primary aim of the study is to investigate the impact of the programme Compassion and Mindfulness Intervention for RA (MITIG.RA) in RA-associated fatigue in comparison with treatment as usual (TAU).

Secondary aims include the effects the intervention has upon the patient’s satisfaction with disease status, overall perceived impact of disease, depression and anxiety levels, psychological flexibility, and self-compassion skills.

### Trial design {8}

The MITIG.RA is a two-arm parallel superiority randomized controlled trial. Participants will be randomized (1:1) into one of two conditions: the experimental condition (MITIG.RA programme plus TAU) and the control condition (TAU only). The first version of the protocol was registered in ClinicalTrials.gov (NCT05389189) in May 2022, before the enrolment of patients. This protocol was written in accordance with the Standard Protocol Items: Recommendations for Interventional Trials (SPIRIT) guidelines. The Consolidated Standards of Reporting Trials (CONSORT) guidelines will be followed when reporting the results of the study.

## Methods: participants, interventions and outcomes

### Study setting {9}

The study is conducted in the Rheumatology Department at Centro Hospitalar e Universitário de Coimbra, an academic rheumatology department in a Portuguese tertiary hospital.

### Eligibility criteria {10}

Participants will be recruited among adult patients with RA followed in the Rheumatology Department at Centro Hospitalar e Universitário de Coimbra.

All consenting patients will be clinically evaluated by the rheumatologist of the research team and fill out the Rheumatoid Arthritis Impact of Disease score (RAID) [54, 55] and the Patient Experienced Symptom State (PESS) [56, 57] to ascertain eligibility.

For patients to be eligible to participate in this study, they must meet the following inclusion criteria: (a) age between 18 and 65 years, (b) meet the 1987 ACR [58] or 2010 ACR/EULAR [59] classification criteria for RA, (c) be currently in PGA-near-remission (as defined by tender and swollen 28 joint counts and CRP (mg/dL) ≤ 1 and a PGA value > 1), (d) a score in the domain fatigue of the ≥ 3, (e) a PESS rating < ‘good’, and (f) under stable medication (at least 3 months).

Participants will be excluded from the study in case they present one or more of the following criteria: (a) less than 6 years of formal education; (b) unable to attend Zoom meetings unaided; (c) unable to complete self-report measures unaided; (d) presence of pain-related comorbidities (e.g. fibromyalgia or osteoarthritis); (e) presence of other comorbid medical conditions that may cause fatigue, such as anaemia (Hb < 10 mg/dL), uncontrolled hypothyroidism, or cancer; (f) presence of severe psychological symptoms or disorders (e.g. psychosis, severe depression, substance abuse); (g) currently undergoing psychological or formal psychiatric treatment; (h) pregnant patients; (i) presence of high levels of disability (advanced articular/bone erosion); and (j) inability or refusal to provide informed consent. Those who decline to participate will be inquired about the underlying reasons.

### Who will take informed consent? {26a}

The potentially eligible patients will be contacted by the research nurse who will explain the study and invite them to participate. Willing patients will receive the informed consent form with detailed information and will have the opportunity to clarify any issue with the rheumatologist from the research team to ensure a full understanding of the entire clinical trial. Only patients who are freely willing to participate and sign the written informed consent will be recruited. Participant’s written consent will be obtained prior to any study procedures.

### Additional consent provisions for collection and use of participant data and biological specimens {26b}

This trial does not involve collecting biological specimens for storage. There are no ancillary studies that require additional consent provisions.

### Interventions

#### Explanation for the choice of comparators {6b}

All patients will be treated with the standard of care (TAU). The difference between arms will be the addition of the Compassion and Mindfulness Intervention for RA.

#### Intervention description {11a}

Development of the intervention the MITIG.RA programme follows the Medical Research Council’s (MRC) framework for the development of complex interventions, last updated in 2019 [60] and was developed by a multidisciplinary team with clinical and academic experience in RA, composed of rheumatologists, nurses, and psychologists. MITIG.RA design was informed by existing protocols of individual and hybrid mindfulness, acceptance, and compassion-based interventions [42, 49] and on the RAFT study [61].

The experimental intervention, described below, will be delivered in a group format, online, for eight consecutive weeks, followed by two booster sessions after 4 and 12 additional weeks.

#### Intervention content

The MITIG.RA programme incorporates the following key topics during the 8 weeks of intervention (1st phase):

(i) Psychoeducation on RA, sleep hygiene, exercise, and general nutritional recommendations (promote behavioural change and self-care, boost the sense of self-worth and self-efficacy)
(ii) Activity engagement and pacing
(iii) The functioning of the mind and its problematic patterns
(iv) Focusing on the ‘here and now’ (mindfulness)
(v) Learning new ways of self-relating—self-compassion
(vi) Making room for suffering (acceptance)
(vii) Moving towards what matters (identification of valued life directions and promotion of consistent values and goal-directed behaviour)
(viii) Promote the implementation of the learned competencies in everyday life; identify and deal with future setbacks

The booster sessions, at 4 and 12 weeks after completion of the first phase, will focus on the revision of previously learned concepts, evaluation of potential barriers/difficulties encountered, strategies employed to deal with them, and clarification of any impending question. They will also revisit and reinforce the continued practice of mindfulness, acceptance, and compassion exercises as daily practices.

An overview of the intervention is provided in Table 1.

**Table 1:** Intervention overview

Session	Module	Content and learning objectives
**Session 1**	Psychoeducation	• Introduction to the programme: goals, overview, and ground rules
		• Participants’ presentation, motivations to participate, and expectations about the intervention
		• Identification of current difficulties in dealing with fatigue, prior coping attempts, and their costs-benefits—creative hopelessness
		• Psychoeducation about RA and fatigue (e.g. ‘drivers’ and ‘feeders’ of fatigue)—the importance of healthy lifestyle habits (sleep hygiene, physical activity, stress, and pacing)
**Session 2**	Psychoeducation and mindfulness skills development	• Psychoeducation about the body-mind link, the function of the mind, and its patterns
		• Promoting mindfulness skills and body awareness • Introducing mindfulness in daily life:
		• informal practice
**Session 3**	Mindfulness skills development	• Promoting mindfulness skills and body awareness
		• Interoceptive exposure
		• Get to know your ‘judgy’ mind
**Session 4**	Promoting acceptance	• Control as a never-ending source of suffering
		• Learning to ‘unhook’ and making room for discomfort
**Session 5**	Self-compassion	• Cultivating compassion towards others, with a special focus on kindness
		• Compassion from others: an ally or an enemy?
		• Exploring new ways of communicating effectively
**Session 6**	Self-compassion and loving kindness	• What is compassion got to do with it—compassion flows and compassion towards the self
		• Bringing compassion to the body
		• Fostering a different form of self-self and self-other relating
**Session 7**	Values and committed action	• Living a meaningful life (values work, identification of ‘drainers’ and ‘energizers’, values-based goals setting)
		• Step-by-step action planning
		• Identification of barriers and facilitators to committed action (reinforce the importance of pacing)
**Session 8**	Closing session	• Review of the key concepts and take-home messages
		• Identification and discussion of potential setbacks and strategies to deal with them
		• Feedback on the intervention and progress made attending to the initial expectations
**Follow-up after 2 and 4 months**	Booster sessions	• Refreshment of the main concepts; evaluate difficulties in the implementation and/or practice of learned skills; revisit and consolidate mindfulness, acceptance, and compassion exercises from the sessions

#### Implementation

A board-certified clinical psychologist with training in contextual approaches will implement the protocol, following the intervention manual. The facilitator will be provided with peer supervision during the implementation of the intervention. The intervention will be implemented in groups of 10 RA patients in 8 × 2 h weekly sessions, followed by 2 × 2 h Zoom booster sessions after 4 and 12 weeks post-intervention. The sessions will be held in private Zoom conferences, allowing the participants to interact and collaborate.

Each session will employ group dynamics, visual aids, comprehensive metaphors, complementary e-books, and experiential exercises. All sessions will follow the same general structure: they will open with a gentle grounding exercise, followed by a brief review of the proposed weekly assignment (feedback, difficulties, and comments on the practices). Then, the main session’s theme and underlying concepts are introduced, followed by the practice and sharing and discussion of the guided experiential exercises. The session closes up with a brief exercise.

The implementation and consolidation of learned strategies in daily routines and practices will be regularly encouraged and monitored. Supporting audio materials featuring guided exercises will be provided for this purpose. A logbook will be used for participants to register notes, homework, and exercises and to promote consciously driven and goal-oriented behaviour.

To be considered an ‘intervention receiver’, each participant will be required to have attended, at least, the first session of the intervention. Participants attending a minimum of 6 sessions will be considered completers. Reasons for drop-out will be assessed and registered whenever possible.

#### Fidelity assessment

Intervention delivery will be performed through online group sessions using an online platform. The presence of patients in the sessions will be registered and rated for fidelity. An ‘effective’ fidelity rate has not yet been established; however, we assume that attending at least 6 sessions is likely to be adequate.

#### Treatment as usual (TAU)

All participants will benefit from the usual standard care in accordance with the current practice at the participating centre, respecting international recommendations for the management of RA. Treatment as usual consists of regular appointments with the accompanying care team, mainly involving disease assessment, treatment adjustments, and lifestyle recommendations. The usual care may include, according to the caring physician’s orientation, treatment of comorbid conditions such as, for instance, depressive or insomnia-related symptoms with the use of antidepressants and benzodiazepines. All medications and adjunctive therapies will be registered at baseline and at each visit during the study period.

#### Criteria for discontinuing or modifying allocated interventions {11b}

Patients who started psychological support outside of the study will discontinue the trial. Patients in the intervention group reporting severe adverse events or experimenting a severe worsening of their clinical condition will discontinue the study and be referred for additional treatment.

Participants may choose to stop participating in the intervention or study for any reason and will be informed on the permission, consent, and assent forms that they can choose to stop participating in the study at any time without consequence.

#### Strategies to improve adherence to interventions {11c}

Participants will be reminded regularly by the research nurse of the online sessions. A complete timetable of online sessions will be provided for each participant at the inclusion. Phone messages and email reminders will be sent 24 h and 1 h before each online session. Similar procedures will be implemented regarding the online assessment battery comprising a set of validated self-report measures at each time point. In case of absence, the patient will receive a phone call from the research nurse within 24 h later.

#### Relevant concomitant care permitted or prohibited during the trial {11d}

Patients enrolled in the study are prohibited from receiving any psychological or formal psychiatric treatment during the trial.

#### Provisions for post-trial care {30}

Following ethical requirements, participants assigned to the control condition (i.e. TAU) will be given the opportunity to receive the newly developed intervention afterwards. Those undergoing the experimental condition (MITIG.RA) will be referred, in case they wish so, to further individual psychological support.

### Outcomes {12}

#### Primary outcome

The following is the primary outcome:

- Fatigue: Fatigue levels will be assessed by the 0–10 numerical rating scale assessing fatigue as part of the RAID [54], described below. Changes at the 3-month follow-up after the second booster session, i.e. 32nd week will be considered.

#### Secondary outcomes

The following is the secondary outcomes:

- Satisfaction with disease status—PESS: The Patient Experienced Symptom State (PESS) [56] evaluates the patients’ degree of satisfaction with their RA status during the last week, through a single item rated on a 5-level Likert scale response (‘very bad’, ‘bad’, ‘acceptable’, ‘good’, and ‘very good’).
- Perceived impact of disease—RAID and RAID.7: The RAID is a 7-item patient-derived measure designed to evaluate the patients’ perceived impact of RA upon important health-related domains, namely pain, functional disability, fatigue, sleep, physical well-being, emotional well-being, and coping. The items are rated using 11-point numeric rating scales. Domains can be combined into a single score (RAID) [55, 62] or used separately (RAID.7) [54]. Higher scores indicate a greater impact of disease.
- Anxiety and depression—HADS: Levels of emotional distress will be measured through the Hospital Anxiety and Depression Scale [63, 64]. This scale comprises 14 items, rated on a 4-point Likert scale, aimed at screening for the presence and severity of anxiety and depressive symptoms in the last 7 days. Higher values are indicative of more severe levels of symptoms, with a cut-off score of 11 being indicative of a clinically significant mood/anxiety disorder.
- Self-compassion—SCS-sv: Self-compassion will be assessed by the Self-Compassion Scale [65, 66]. This 12-item measure is a shorter form of the original scale developed by Neff [67] and aims to assess the type of relationship one establishes with oneself in the face of setbacks or difficult times. Items are rated on a 5-point Likert scale, with greater values indicating greater levels of self-compassion. While several factorial solutions have been proposed, in this study, we will use a two-factor structure, comprising the self-compassionate attitude subscale and the self-critical attitude subscale.
- Psychological flexibility—CompACT: Psychological flexibility, a key construct in ACT, will be assessed through the Comprehensive Assessment of Acceptance and Commitment Therapy (CompACT) [68]. This is a 23-item self-report measure rated using a 7-point response scale from 0 (‘strongly disagree’) to 7 (‘strongly agree’). The scale comprises 3 factors, namely openness to experience (e.g. ‘I can take thoughts and feelings as they come, without attempting to control or avoid them’), behavioural awareness (e.g. ‘I do jobs or tasks automatically, without being aware of what I am doing’), and valued action (e.g. ‘I can identify the things that really matter to me in life and pursue them’). Scores range from 0 to 60 (openness to experience subscale), 0 to 30 (behavioural awareness subscale), 0 to 48 (valued action subscale), and 0 to 138 (total score) and are computed by summing all respective items. Higher scores indicate greater psychological flexibility.
- Patient global assessment of disease activity—PGA: In our study, the PGA will not be used as an outcome measure but as a tool used to define eligibility. PGA is one of the most widely used patient-reported outcomes in RA and is found in several scores, such as the 28-Joint Disease Activity Score (DAS-28). The PGA is a holistic assessment of disease that goes beyond the objective measures of inflammation (acute phase reactants) and/or structural damage (radiographic) [66, 69]Clique ou toque aqui para introduzir texto.. Higher scores represent a higher level of disease activity or a worse global health, since the proposed definition of ‘low global assessment’ is ≤ 2.0 on a 0 to 10 scale [70].
- Safety of the intervention: Safety-related outcomes will be evaluated through participants’ reporting of adverse events and will include information regarding the nature/type of adverse event, duration and frequency of the event, and degree of association with the intervention (‘no’, ‘probably’, ‘possibly’, ‘yes’).
- Feasibility and acceptability: Feasibility will not be formally assessed in this study as they seem well-established in the literature regarding similar interventions in similar contexts [51, 61]. However, all aspects related to these dimensions will be proactively monitored and registered, namely through rates of attendance and drop-out and the respective underlying reasons.

#### Participant timeline {13}

**Fig. 1:**
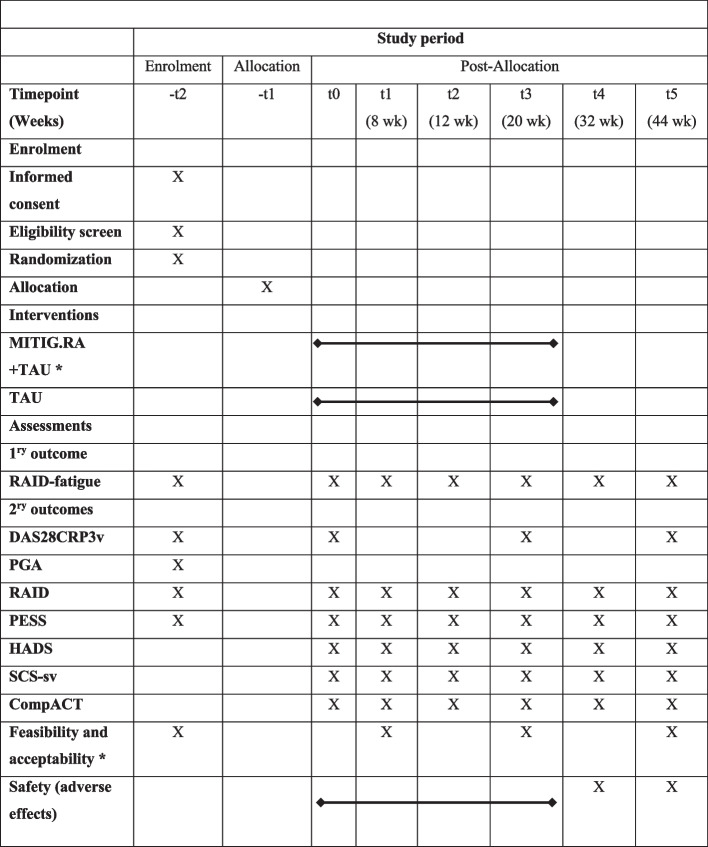
Schedule of enrolment, intervention, and assessments. Legend: *only for the experimental (active) condition. Booster sessions at 1- and 3-month follow-ups are considered as part of the intervention: T0 = baseline; T1 =post-intervention; T2 = before 1st booster session; T3 = before 2nd booster session; T4 = 12-week follow-up; T5 = 24-week follow-up. MITIG.RA, Compassion and Mindfulness Intervention for Rheumatoid Arthritis; TAU, treatment-as-usual; RAID, Rheumatoid Arthritis Impact of Disease; DAS28CRP3v, Disease Activity Score using 28 joints and C reactive protein and three variables; PGA, Patient Global Assessment of disease activity; PESS, Patient Experienced Symptom State; HADS, Hospital Anxiety Depression Scale; SCS-sv, Self-Compassion Scale; CompACT, Comprehensive Assessment of Acceptance and Commitment Therapy

See Fig. 1 for the SPIRIT figure with the participant timeline.

#### Sample size {14}

The primary outcome of the study will be the difference in fatigue scores between the two intervention groups at week 32, controlling for baseline values. Based on the minimum clinical improvement established for RAID score and on our previous study assessing the responsiveness of RAID.7i, we expect a minimum difference in fatigue scores between the two groups of at least 3 points [54, 71, 72].

A sample size of 91 was indicated by G*Power for analysis of covariance (ANCOVA) [*α* = 0.05, 90% statistical power, effect size of 0.50]. Assuming a 30% attrition rate and rounding up for convenience, we plan to recruit a total of 120 participants (60 participants per arm).

#### Recruitment {15}

Patients will be recruited from the rheumatology outpatient clinic. Currently, more than 1300 RA patients have regular follow-ups in our department, 700 being below the age of 65 years, and around 40% are in PGA-near-remission. All rheumatologists from our department will be informed about the study, and a kind reminder will be sent every week. Additionally, our research nurse will verify the patients’ registry to identify potential candidates to participate in the study.

### Assignment of interventions: allocation

#### Sequence generation {16a}

After baseline assessment, participants will be assigned either to the intervention or the control condition with a 1:1 allocation by means of a computerized random number generator (www.random.org) stratified by depression score (above/below HADS score of 8) [63].

#### Concealment mechanism {16b}

Allocation sequence concealment will be guaranteed by using a third party (a research member responsible only for generating the randomization schedule) and by using sealed envelopes that are opened only after patients’ enrolment.

#### Implementation {16c}

Recruiters will engage with potential participants. After the eligibility criteria have been confirmed, the allocation sequence will be generated by a computerized random number generator by the coordinator of the study (www.random.org). Another team person will assign participants to the intervention (either MITIG.RA or TAU) according to those sequences and subsequently enrol participants in the study.

### Assignment of interventions: blinding

#### Who will be blinded {17a}

Given the nature of the study, it will be impossible to blind participants regarding their allocation. However, the following types of blinding will be implemented: blind assessment (the rheumatologist performing the examinations will be blind to the patient’s allocation) and blind analysis (the researcher performing the statistical analysis will be blind to participants’ allocation).

#### Procedure for unblinding if needed {17b}

There will be no unblinding procedures due to the nature of the intervention.

### Data collection and management

#### Plans for assessment and collection of outcomes {18a}

The research team is responsible for the assessment and collection of outcomes, being the content and timing of the assessment similar to both intervention groups.

Clinical data and outcome measures will be collected online, via Reuma.pt (www.reuma.pt), the official and ethically approved national register of rheumatic patients.

Sociodemographic information will be collected from the patients’ files at baseline. Measurement of C-reactive protein levels and clinical examination and assessment of disease activity will be performed and registered at screening, baseline, and after 20 and 44 weeks by the rheumatologist. Medication monitoring and recording will be performed throughout the study, from screening to the last follow-up period.

At baseline, 8 weeks (post-intervention), 12 weeks (before the 1st booster session), and 20 weeks (before the 2nd booster session), participants will complete an online assessment battery comprising a set of validated self-report measures (see Fig. 1). This assessment will be repeated at 32 and 44 weeks to evaluate the persistence of potential therapeutic gains over time. Each patient will have private access to the questionnaires through a personal registered account. The research nurse will determine the correct completion of all measures. Those responsible for delivering the interventions will not be involved in any form of data collection and analysis over the duration of the trial.

#### Plans to promote participant retention and complete follow-up {18b}

The research nurse will contact the participants to remember online scheduled sessions according to the protocol, as well as the online assessment battery comprising a set of validated self-report measures at each time point defined in the protocol.

The correct completion of all measures will be monitored by a research nurse.

#### Data management {19}

In this RCT, all data will be gathered online and recorded automatically. Data will be stored in a secure server, Reuma.pt, which works as an electronic clinical file available, designed to be used in real time while observing the patients. Patients have access to a dedicated and protected area where they can fill in the self-assessment questionnaires. Their answers are automatically loaded on the consultation page. Reuma.pt is registered with the national data protection authorities and respects all applicable ethical principles and the General Data Protection Regulation.

A specific protocol for MITIG.RA trial will be developed and protected by personal and non-transmissible username and password, limited to the research team and for the included patients.

The data entry screens will resemble the paper forms of the self-questionnaires included in the trial. Data integrity will be enforced through a variety of mechanisms. Referential data rules, valid values, and range checks are considered in the Reuma.pt. The option to choose a value from a list of valid codes and a description of what each code means is available where applicable. Additional errors, missing data, or inconsistencies will be detected by Reuma.pt, and a list of irregularities/per patient will be generated and shown to the research nurse whenever the patient profile is accessed in the platform.

After study completion, anonymized data will be made available for statistical analysis.

#### Confidentiality {27}

Subject confidentiality is strictly held by the participating researchers. All source records, including electronic ones, will be stored in secured systems. All data in Reuma.pt will be encrypted, anonymized, and made accessible only to the research team, through personal and untransmissible username and password.

No identifiable information concerning enrolled subjects will be released to an unauthorized third party. Subject confidentiality will be maintained across all submission and publishing stages.

#### Plans for collection, laboratory evaluation, and storage of biological specimens for genetic or molecular analysis in this trial/future use {33}

C-reactive protein (CRP) collection will be performed as part of the routine assessment and care of patients with RA, in order to determine disease activity status. No other biological specimens will be collected or stored in the scope of this trial.

## Statistical methods

### Statistical methods for primary and secondary outcomes {20a}

Descriptive analysis and test differences will be used to compare the baseline demographics and participants’ characteristics as well as variables of interest, such as participant’s dropout rates, reasons for dropout, and the presence/absence and severity of negative effects.

An intention-to-treat analysis will be conducted whenever possible and complemented with per-protocol analysis if needed.

Preliminary analyses will be conducted to guarantee that the necessary assumptions for the following tests are met. Analysis of covariance (ANCOVA) will be used to assess between-group differences in fatigue scores (the primary outcome) at the 3-month follow-up after the second booster session (32nd week), controlling for baseline values. The same analytic procedure will be used to examine the between-group differences in perceived disease activity, depression, mindfulness, and self-compassion (secondary outcomes) in the 32nd week. All effect sizes will be reported.

Additionally, time × group interaction effects on fatigue levels, perceived disease activity, depression, mindfulness, and self-compassion scores across the different time points will be tested via repeated measures analysis of variance (ANOVA), within-between interaction (with Bonferroni correction).

Potential predictors of treatment response (e.g. sociodemographic factors, baseline levels of fatigue, disease impact, depression, and anxiety) will be explored through regression analyses.

### Interim analyses {21b}

No interim analysis of the variables of interest is planned.

### Methods for additional analyses (e.g. subgroup analyses) {20b}

Not applicable.

### Methods in analysis to handle protocol non-adherence and any statistical methods to handle missing data {20c}

Missing data analysis will be performed to determine the presence and level of randomness of missing data. Multiple imputation will be used to handle the missing data [73].

### Plans to give access to the full protocol, participant-level data, and statistical code {31c}

Not applicable.

### Oversight and monitoring

#### Composition of the coordinating centre and trial steering committee {5d}

The trial will be directed by the leading investigators (JAPS and CD). There is no coordinating centre or trial steering committee as part of this trial. The leading investigators will supervise the work of the research team. Regular project meetings with the research team will be conducted to provide continuous updates on staff activities, problem solve, and provide a forum for project coordination.

#### Composition of the data monitoring committee, its role, and reporting structure {21a}

The data monitoring committee (DMC) was considered not needed considering being a single centre involved in the study, the type of intervention, and the low risk of the study.

The leading investigators will meet weekly to discuss the status of the research and any possible unforeseen issues. The research team will report any issues that arise during the trial to the leading investigators.

#### Adverse event reporting and harms {22}

This is a low-risk study. We do not anticipate any adverse events or risks for the participants. Nevertheless, any form of unfavourable event will be evaluated and reported.

#### Frequency and plans for auditing trial conduct {23}

The monitoring process will not be independent from investigators, and the research team will be responsible for conducting and monitoring the clinical trial. All project staff members will meet regularly in order to monitor the protocol, resolve issues regarding the project, and make sure that participant safeguards are constantly being properly maintained.

The research nurse will scan the incoming data on a regular basis to ensure that no identifying information has been shared in the open-ended fields and will be instructed to record any problems seen or concerns about any participants.

External auditing process is not planned.

#### Plans for communicating important protocol amendments to relevant parties (e.g. trial participants, ethical committees) {25}

In case of any protocol amendment, all modifications must be approved by the Ethics Committee of the Centro Hospitalar e Universitário de Coimbra.

#### Dissemination plans {31a}

The trial is currently registered on ClinicalTrials.org under the reference number NCT05389189. Results obtained will be disseminated on a national and international level in the form of conference presentations and scientific articles published in relevant peer-reviewed journals.

## Discussion

By combining components of contextual CBT interventions, we have designed a new psychosocial intervention aiming at the management of fatigue in RA patients. Fatigue is recognized as a frequent and important manifestation of RA which is typically very difficult to manage in practice. Although fatigue constitutes our primary endpoint, other relevant related domains, including the impact of disease, depression, and anxiety, will also be assessed and addressed and, hopefully, improved.

Although classical CBT interventions have been found to be useful in the past, effect sizes are relatively small and considered unsatisfactory [26, 27, 61, 74, 75].

The interventions’ core comprises practices of mindfulness, acceptance, and compassion-based interventions, designed to promote relevant changes in the bidirectional interplay between physical and psychological experiences endured by patients with RA. The key strength of the intervention is found in the emerging validity of CCBT interventions in diseases where body and mind are both potentially and interactively affected [37, 76–78]. A group format is not only cost-effective in nature [79] but also recognized as a powerful element in interventions focused on coping, chronic pain, mood, and disability [80]. At the same time, an online delivery method may be particularly suitable for people who due to accessibility, mobility, financial, or chronic-related disability issues are often unable to enrol at in-person modalities [35].

This study features a number of strengths. The study is focused on the management of fatigue in RA patients, which is challenging in clinical practice, demanding more research on effective interventions. The study is based on a solid methodology, applies a randomized controlled trial, and involves an experienced multidisciplinary team.

The repeated assessment of outcome measures at different moments in time will allow us to evaluate the efficacy and usefulness of the intervention and the booster sessions and the persistence of potential therapeutic gains up to 6 months after the end of the intervention.

Some limitations should also be recognized. It is a unicentric study which limits the generalization of our results. Online intervention increases the risk of selection bias, as only participants with internet access and minimal technological aptitudes will be able to participate. Also, the patient’s perception of an online intervention regarding the quality of the therapeutic relationship might be questionable, a concern frequently shared by therapists [81]. In further editions, a more inclusive approach might be employed. These factors emerge in addition to those typically recognized in all psychotherapy interventions, namely their dependence on non-specific factors and on patient’s features, motivation, and adherence [82].

In addition, the age limited to 65 years is restrictive in nature but necessary in our social setting, as older ages are associated with a high prevalence of technological and health illiteracy and a greater number of health comorbidities.

Another limitation is that the main outcome data is based on self-report, using a NRS of fatigue. However, RAID.7i-fatigue NRS showed to be a valid, feasible, reliable, and responsive instrument avoiding the burden for clinical practice of more complex and time-consuming instruments.

The results of this study will be of great relevance for daily clinical practice. Our findings will highlight the role of CBT in the management of severe fatigue in patients with RA in inflammatory remission, promoting its inclusion in RA management. Moreover, the web-based delivery may facilitate easy access to CBT to larger groups of the population worldwide. Therefore, our study may.

In conclusion, the MITIG.RA is a tailored intervention merging contextual-cognitive behavioural components aimed at improving fatigue, depression, anxiety, impact of disease, and self-regulation processes in RA.

## Trial status

MITIG.RA, v2 Protocol. May 22.

At this stage, no patient was enrolled. The recruitment will be expected to start in October 2023. The recruitment is estimated to be completed in February 2024.

## Data Availability

All data collected in the context of this study will be made available to external researchers upon reasonable request for cooperative investigation.

## Abbreviations

RA: Rheumatoid arthritis
T2T: Treat-to-target
EULAR: European Alliance of Associations for Rheumatology
ACR: American College of Rheumatology
PGA: Patient global assessment
CBT: Cognitive behavioural therapy
ACT: Acceptance and commitment therapy
MITIG.RA: Compassion and Mindfulness Intervention for RA
TAU: Treatment as usual
DAS283v-CRP: Disease Activity Score using 28 joints and C reactive protein and three variables
PESS: Patient Experienced Symptom Status
RAID: Rheumatoid Arthritis Impact of Disease
RAID.7: Rheumatoid Arthritis Impact of Disease 7 items
Hb: Haemoglobin
HADS: Hospital Anxiety and Depression Scale
SCS-sv: Self-compassion Scale – short version
CompACT: Comprehensive Assessment of Acceptance and Commitment Therapy
ITT: Intention-to-treat
CRP: C-reactive protein
